# Emotion Regulation Compensation Following Situation Selection Failure

**DOI:** 10.1038/s41598-018-23654-2

**Published:** 2018-04-03

**Authors:** Lara Vujović, Heather L. Urry

**Affiliations:** Psychology Department, Tufts University, Medford, MA 02155 USA

## Abstract

We conducted two within-subjects experiments to determine whether people use alternative emotion regulation (ER) strategies to compensate for failure of situation selection, a form of ER in which one chooses situations based on the emotions those situations afford. Participants viewed negative and neutral (Study 1, N = 58) or negative, neutral, and positive pictures (Study 2, N = 90). They indicated for each picture whether they wanted to terminate presentation (Study 1) or view it again (Study 2). We manipulated the outcome of this decision to be congruent with participants’ wishes (success) or not (failure), and measured self-reported ER strategies and emotional responses. Although participants terminated negative situations more often than neutral situations (Study 1), or chose to view positive pictures more frequently than neutral, and neutral more frequently than negative (Study 2), there was little evidence of compensation in the wake of situation selection failure. Overall, we conclude that although people choose situations based on affect (i.e., attempt to end or avoid high-arousal negative situations and pursue high-arousal pleasant ones), they do not generally use the alternative ER strategies that we assessed (rumination, reappraisal, distraction) to compensate when the situations they select fail to materialize in this experimental context.

## Introduction

We routinely make choices to approach or avoid situations in part based on how we think those situations will make us feel^1^. Selecting a situation based on its presumed emotional potential is one form of emotion regulation (ER), a set of processes that impacts which emotions one experiences, when, and how one experiences and/or expresses them^2^. It is important to study situation selection because this form of ER is often effective, especially among people who have difficulties regulating their emotions otherwise^3^, and because situation selection can have a significant impact on well-being. For instance, people often eat unhealthy food, consume alcohol, or avoid anxiety-provoking situations thinking it will make them feel better^4–7^. Although such activities will feel good in the moment, indulging in them too frequently puts people at risk for overeating, overdrinking, and excess anxiety, all of which have consequences for their health. Understanding the factors that explain why and how people select situations based on how they want to feel may, thus, yield insights that improve well-being.

Although understanding why and how people choose situations based on their emotional potential has garnered a fair amount of research, such efforts have provided mixed results thus far. To date, most studies have focused on whether the valence and arousal of available situations guide people’s situation selection. In a laboratory setting, this often involves examining participants’ willingness to spend time looking at digital pictures varying in emotional content. One such study found that participants chose to look at negative pictures longer than at positive pictures^8^. In contrast, a study using a similar paradigm with neutral and negative pictures (no positive pictures) did not find evidence that participants chose to end presentation of negative pictures more frequently than neutral pictures^9^. Rather, participants in this study chose to end high-arousal negative and low-arousal neutral picture presentations more frequently than low-arousal negative and high-arousal neutral pictures, respectively^9^. While the specific pattern varied, both of these studies provided evidence that people select situations in part based on affect, i.e., the degree to which the pictures were pleasant or unpleasant and arousing in nature.

Another method that has been used to study situation selection in a lab setting is the affective environment^10^, which is an enclosed room where people have a limited amount of time (typically 15 minutes) to interact with information (magazines, videos, films etc.) that is positive, neutral, or negative in valence. Here, results are mixed as well: one study found preference for viewing negative and positive stimuli more than neutral^10^, another found a preference for positive situations over negative and neutral^11^, yet another study found a preference for neutral, compared to negative and positive material^12^. The mixed nature of these findings could be attributed to the role of arousal: whereas explicitly examined in only one study^10^, arousal of the stimuli had an effect on the number of selected situations regardless of their valence. Although the specific pattern varied, these studies, just like the ones examining willingness to spend time looking at pictures, supported the idea that people choose situations based on emotional potential.

Regardless of the degree to which people choose situations based on valence and arousal, attempts to choose situations that make us feel how we want to feel do not always succeed; circumstances beyond our control may block our access to a desired situation. Yet, according to the Selection, Optimization, and Compensation with Emotion Regulation (SOC-ER) framework, we might still be successful in regulating our emotions if we compensate by employing other ER strategies^13,14^. For example, to combat feeling sad or anxious, we might make lunch plans with a dear friend; if those plans fall through, we can select a new situation (e.g., call the friend by phone) or an entirely new strategy (e.g., reappraise the situation that is making us feel sad or anxious, distract ourselves with neutral thoughts). Without such compensatory manoeuvres, situation selection failure might give way to the original emotions (sadness or anxiety, in this example). It might also prompt additional negative (and reduce positive) emotions as a general consequence of failing to satisfy a goal^15^.

Although the notion of compensation for ER failure makes intuitive sense, there are just a few studies supporting the idea, sometimes indirectly. For example, in one study, participants who failed to reduce negative emotional experiences using cognitive reappraisal turned to avoidance; those who were instructed to use acceptance turned to avoidance less^16^. In addition, in another study, even when instructed to use cognitive reappraisal, participants often used uninstructed ER strategies, particularly in higher-intensity situations^17^. People also switched strategies based on emotional internal feedback, including subjective awareness of emotion and related physiological processes indicating the success or failure of the current ER strategy^18^. While these examples suggest that people try to compensate for ER failure, studies are needed that experimentally manipulate success or failure of an ER strategy to test whether this manipulation results in greater use of alternative ER strategies. To our knowledge, only one study^9^ has done so. In that study, we found no evidence of compensation. This absence of evidence may indicate that compensatory ER simply does not occur, at least not in the experimental context within which we looked for it. It might also reflect a limitation of that study, namely that we assessed only one alternative ER strategy, overt attentional deployment via eye tracking.

In sum, while previous work suggests varied specific patterns, it is clear that people choose situations based on emotional potential, in particular based on valence and arousal, and that the tasks in which participants manage their viewing of emotional pictures effectively capture situation selection in a lab setting^8,9^. Whereas research suggests that people tend to use multiple ER strategies to improve how they feel^16,18^, which could reflect attempts to compensate for initially unsuccessful ER, there is currently no evidence of compensation following manipulations of ER failure. Thus, in this paper, we present two studies for which the goal was to provide participants the opportunity to choose situations that vary in affective tone, and determine whether they engage in compensatory ER when their attempts to choose situations based on emotional potential fail by design. In Study 1, we used a variant of the task used in our previous work^9^ to assess several possible compensatory ER strategies. The primary goal was to determine whether participants would use alternative ER strategies to compensate for ER failure. In Study 2, we sought to overcome some design limitations of Study 1, extend the emotional context of our previous work by including positive stimuli, and study situation selection and ER compensation in a larger on-line community sample. Our materials and data for both studies are publicly available at https://osf.io/e9wfh.

## Study 1

In this study, our goal was to determine whether participants would use alternative ER strategies to compensate for situation selection failure. Participants viewed high-arousal negative and low-arousal neutral pictures. As our measure of situation selection, participants were told they could press a button if they wished to stop looking at each picture. We manipulated the outcome of pressing the button; on 70% of the trials, the button press was successful in ending picture presentation and, on 30% of the trials, the button press failed to end picture presentation. To assess compensatory ER, after each trial, we presented participants with a multiple-choice question asking them to indicate which of three ER strategies they used: rumination, distraction, and/or reappraisal. Emotional responses were assessed by corrugator, heart rate (HR), and electrodermal activity, as well as the subjective ratings of negative emotion and motivation to change emotion.

We hypothesized that one of two alternatives might be observed after the success/failure outcome. On the one hand, people may experience more negative emotion in the failure condition relative to the success condition, as reflected in self report and changes in physiology (Hypothesis 1a). On the other hand, they may compensate by engaging in other ER strategies. In this case, negative emotion would be similar in the failure and success conditions, but there would be greater compensatory ER on failure relative to success trials (Hypothesis 1b), as reflected in self-report. It was possible that this pattern would be moderated by the affective nature of the stimuli (negative, neutral). However, given that our previous work showed that both low-arousal neutral and high-arousal negative pictures exhibited high, equivalent rates of button pressing to make the picture disappear, it was also possible that the outcome manipulation would affect the negative and neutral trials similarly^9^. To account for variation in responding across trials, we used multilevel analyses to test these hypotheses.

## Method

### Participants

Our goal was to have approximately 50 usable observations in hypothesis testing, which would give us 80% power to detect effect sizes of Cohen’s dz ≥0.36. We anticipated losing data due to equipment problems, artefacts, and/or extreme or missing values, thus ultimately we recruited 58 Tufts University undergraduates who participated in exchange for course credit. No participants were excluded from the analyses. Of those participants, 39 were female (67.2%), 46 were White (79.3%), nine were Asian (15.5%), one was African American (1.7%), and one person did not disclose racial/ethnic information (1.7%); demographic data for one person were missing. The age range was from 17 to 22 years (M = 19 years, SD = 0.97 years). All procedures were approved by the Social, Behavioral, and Educational Research Institutional Review Board at Tufts University and the Army Human Research Protections Office, and all methods were performed in accordance with their guidelines and regulations. Participants provided written informed consent before participating.

### Materials and Procedures

#### Stimuli

Participants viewed a set of 68 digital colour pictures (800 pixels × 600 pixels) selected from the International Affective Picture System (IAPS)^19^. We established two comparable sets of 34 stimuli: high-arousal negative and low-arousal neutral pictures. The high-arousal negative pictures were highly unpleasant (M = 2.00, SD = 0.46, on a scale ranging from 1 to 9, where 9 = completely happy), and highly arousing (M = 6.74, SD = 0.41, on a scale ranging from 1 to 9, where 9 = completely aroused). The low-arousal neutral pictures were neither pleasant nor unpleasant (M = 5.13, SD = 0.47), and low in arousal (M = 3.08, SD = 0.29). The catalogue numbers of pictures from the IAPS 2008 set used in this study can be found in Supplementary Information.

#### Picture Task

After participants signed the consent form, we calibrated eye tracking for each participant. Bilateral eye-tracking data were collected using a Tobii T120 Eye Tracker (Danderyd, Sweden; sampled at 60 Hz). These data were processed offline using Tobii Studio software. We planned to use eye tracking to measure overt deployment of attention to key information in the pictures. However, due to technical problems aligning data with stimulus events, eye tracking data for this study were not analysed. For details on eye tracking data collection parameters please refer to our previous paper^9^.

They then viewed the above set of 68 pictures, evenly distributed across four blocks. The order in which the 68 pictures were presented was randomized for each participant. Participants were told in advance that they can press the space bar whenever they wished to stop looking at a picture. They were told that most of the time the picture will go away when they press the button, but could also occasionally remain on the screen. The picture task was presented using E-Prime 2.0 (Psychology Software Tools, Pittsburgh, PA).

**Figure 1:**
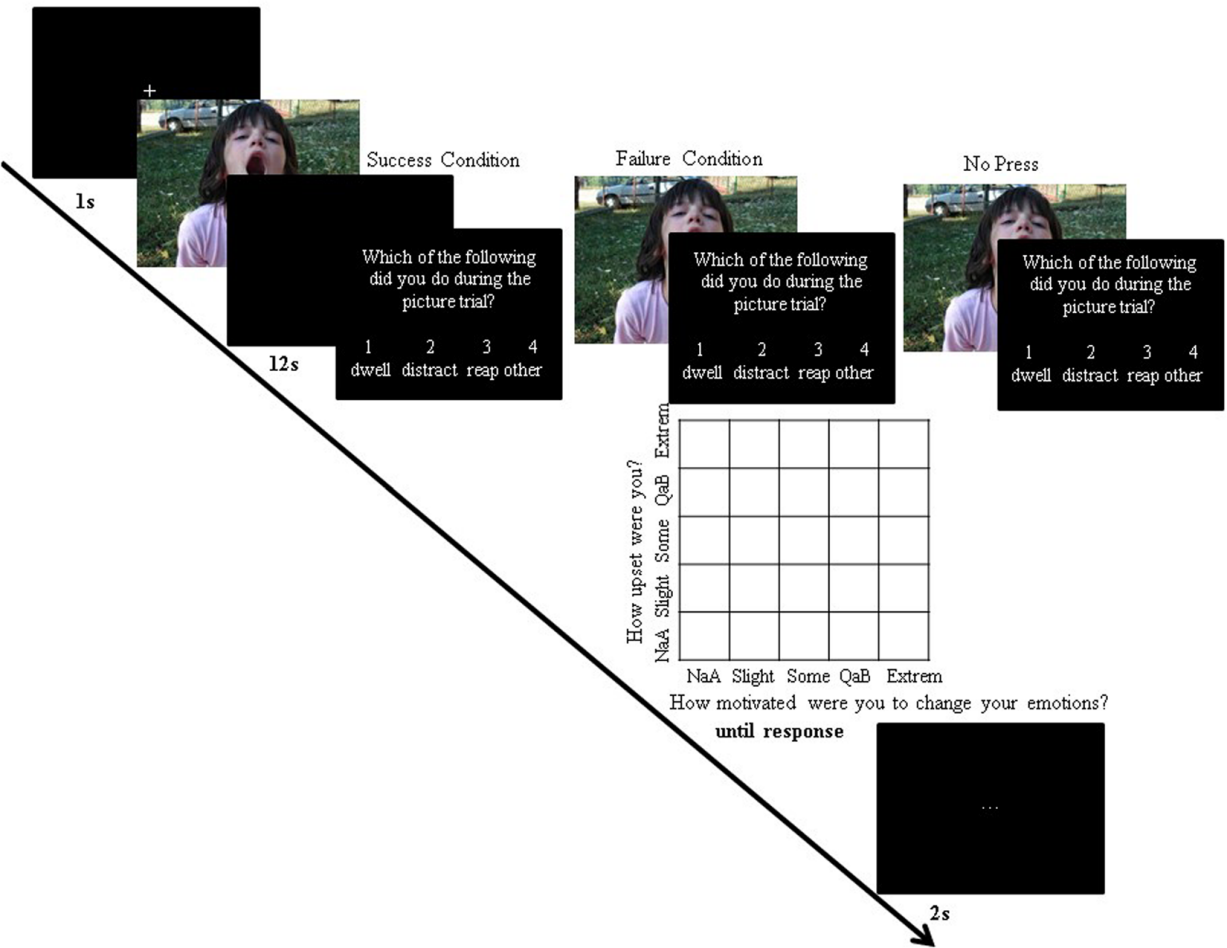
Trial structure for the picture task in Study 1. Whereas we used IAPS pictures for this task, the example picture in this figure is from one of the author’s private collection.

All trials began with a white fixation cross presented in the centre of a black screen for 1 s. The fixation was followed by the presentation of a picture for up to a total of 12 s. Pretesting conducted previously^9^ identified 12 s as the optimal picture presentation time in this context, meaning it yielded a press rate that left room for variation, both higher and lower, as a function of the failure manipulation. If participants pressed the space bar, the picture either: (1) left the screen and was replaced with black screen until a total of 12 s had elapsed (“press success” condition; approximately 70% of the press trials), or (2) remained on the screen until a total of 12 s had elapsed (“press failure” condition; approximately 30% of the press trials) (see Fig. 1).

After 12 s elapsed, participants indicated which ER strategy they used. They could choose none, one, two, or all three of the following: rumination (thought about the content of the picture and how it made them feel), distraction (thought about something other than the picture), and reappraisal (thought differently about/reinterpreted the picture). Participants saw this question on screen: “Which of the following did you do during the picture trial? Select all that apply by pressing the appropriate number on the keyboard: (1) dwelled on the picture content; (2) distracted myself with other thoughts; (3) thought differently about the picture; (4) none of these.” The ER strategy screen remained present until participants responded.

Prior to the picture task, participants completed a training procedure in which they learned what it meant to dwell on the picture content, distract themselves, or think differently about the picture. We provided a sample IAPS picture of a man aiming a riffle at a polar bear, and we described ways in which different ER strategies might be used. For instance, for “distracted myself with other thoughts” an example was: “thought about something that happened to you on the way here”. There were also 10 practice trials to make sure the participants understood the task and the distinctions between these different strategies (see Supplementary Information).

After reporting which ER strategies they used, participants provided a rating of negative affect and motivation to change their emotion via a variant of the evaluative space grid (ESG)^20^, allowing us to efficiently collect two independent ratings. Participants chose one number in a 5 × 5 grid representing their rating for “How upset were you?” (on the X axis) and “How motivated were you to change your emotions?” (on the Y axis) (see Fig. 1). The answer options were: Not At All, Slightly, Somewhat, Quite a Bit, and Extremely. The rating screen remained present until a response was recorded. Lastly, participants saw a black screen with a central white ellipsis that lasted 2 s to provide a brief break between trials.

At the end of the study session, participants completed self-report questionnaires via Qualtrics, assessing exposure to stressors, the Life Experiences Survey^21^, the perceived probability and persistence of a list of emotional states, and the Emotion Reactivity and Perseveration Scale, modelled after the Anxiety Reactivity and Perseveration Scale^22^. They also provided demographic information and information about their horror film watching habits (liking and frequency). The constructs measured in these questionnaires were included as exploratory moderators of observed effects; analyses using these measures are not reported in this paper. After completing this survey, participants were debriefed, compensated, thanked for their participation, and dismissed.

### Independent Variables

We manipulated two independent variables in our picture task. One was type of affect with two conditions (negative or neutral.) The other was the manipulated outcome of trials on which participants pressed the spacebar to terminate picture presentation; this also had two conditions (press failure or press success).

### Dependent Variables

#### Behaviour and Self-Report

To measure situation selection, we recorded **button pressing behaviour** (1 = press, 0 = no press). To measure the speed with which participants chose to end the situation, we measured **button press response time** (in seconds) on trials on which the button was pressed. To measure **compensatory ER** use, there were three variables of interest: self-reported use of rumination, distraction, and reappraisal (1 = strategy endorsed, 0 = strategy not endorsed). To measure the experience of **negative affect** and **motivation to change emotions**, we used participant ratings from 1 to 5 (‘not at all’ to ‘extremely’).

#### Peripheral Physiology

We recorded peripheral physiological data (electrocardiography facial electromyography (corrugator), and electrodermal activity) continuously during the task using an MP150 system (Biopac, Goleta, CA, USA). These data were processed offline using ANSLAB^23^ to capture heart rate (HR), activity over the corrugator muscle (“inner eye brow”) region, and skin conductance level, respectively, during the 12-s window of interest. We divided this time period into pre- and post-button press variables for each trial for each participant, allowing us to capture change in physiology as a function of the attempt to end the situation by pressing the button. For details of data collection parameters, please refer to our previous paper^9^.

### Data Analysis and Retention

We conducted multilevel analyses with two levels using full information maximum likelihood estimation with robust standard errors in Mplus v. 7.11^24^. Continuous predictors were grand-mean centred. For analyses with continuous criterion variables, we estimated predictors as random effects that could thus vary between subjects. However, for analyses with categorical criterion variables, we estimated predictors as fixed effects because the models could not converge otherwise. We applied Bonferroni correction to determine significance levels based on the number of comparisons conducted for each hypothesis test.

Twelve participants never pressed the space bar in response to a picture, thus they had no scores on pre- and post-physiological variables and contributed no scores to the reaction time variable. This essentially reduced our N to a maximum of 46 for all analyses that depended on button pressing, including those in which the manipulated outcome was a predictor. Additional data were lost due to equipment malfunction (e.g., movement artefacts for physiological measurements). The number of participants for each of the hypothesis testing analyses can be found in tables providing inferential statistics.

## Results

### Summary of Preliminary Analyses

In preliminary analyses, participants evidenced increased self-reported negative affect, greater motivation to regulate their emotions, and a decrease in heart rate for negative relative to neutral pictures; there was no such difference in corrugator or electrodermal activity. In addition, they pressed the space bar more frequently on negative relative to neutral trials with no difference in how fast they pressed it. Thus, as expected, the negative pictures garnered larger emotional responses; they also garnered more frequent attempts to terminate the situation. Statistical results of these analyses can be found in Supplementary Information.

### Hypothesis Testing

#### Test of Hypothesis 1a. Did ER failure prompt greater negative emotion relative to ER success, regardless of type of affect?

We conducted four two-level models in which we regressed one of the three physiological measures or self-reported ratings of negative affect (NA) on a type of affect contrast (negative [1] vs. neutral [−1]), a manipulated outcome contrast (failure [1] vs. success [−1]), and an interaction contrast reflecting the product of the affect and manipulated outcome contrasts. To isolate variation in post-press emotional responding, mean activity occurring after button press was regressed on mean activity occurring before button press for each of the three physiological measures. After applying a Bonferroni correction, our critical p-value changed to 0.0125 (0.05/4 criterion variables).

Neither corrugator activity nor electrodermal activity were sensitive to the difference between negative and neutral pictures prior to button press; we, thus, did not analyse those variables further. We also did not have an a priori hypothesis about the role of self-reported motivation post failure, so this variable was not analysed further. Thus, focusing on NA and HR activity, we found that post-press HR deceleration was greater in the failure relative to the success condition, B =−0.59, 95% CI [−0.86, −0.33], p < 0.001. However, people did not report experiencing NA more on failure compared to success trials, B = 0.03, 95% CI [−0.01, 0.07], p = 0.152. Overall, there was some evidence of situation selection failure prompting greater negative emotion relative to situation selection success, regardless of affect of the available situations. Inferential statistics are reported in Table 1, and descriptive statistics are reported in Table 2.

**Table 1:** Inferential statistics (B, SE, 95% Confidence Intervals, and p-values) from the Two-Level Analyses Examining Effects of Valence, Outcome, and their Interaction on Experience of Negative Emotion after Button Press in Study 1.

	Self-reported negative affect			Heart rate			Corrugator activity			Electrodermal activity		
	Estimate	SE	95% CI	Estimate	SE	95% CI	Estimate	SE	95% CI	Estimate	SE	95% CI
Valence	1.18***	0.06	[1.06, 1.31]	−0.62***	0.17	[−0.95, −0.30]	0.05	0.16	[−0.27, 0.37]	0.04	0.03	[−0.02, 0.10]
Outcome	0.03	0.02	[−0.01, 0.07]	−0.59***	0.14	[−0.86, −0.33]	0.02	0.15	[−0.28, 0.32]	0	0.02	[−0.04, 0.03]
Valence*Outcome	0.02	0.02	[−0.02, 0.06]	−0.33*	0.14	[−0.59, −0.06]	−0.03	0.10	[−0.22, 0.16]	0.01	0.01	[0, 0.02]
Model fit												
AIC	4616.42			8555.27			11113.96			277.68		
Free parameters	9			10			10			10		
*N*	46			42			44			43		

Note *p < 0.05, **p < 0.01, ***p < 0.001.

**Table 2:** Parameter Estimates (B and SE) from the Two-level Analyses Examining Effects of Valence, Outcome, and their Interaction on Experience of Negative Emotion after Button Press in Study 1.

	Self-reported negative affect		Heart rate		Corrugator activity		Electrodermal activity	
	Estimate	SE	Estimate	SE	Estimate	SE	Estimate	SE
Valence								
Negative	2.57	0.13	75.94	1.00	9.47	0.62	11.99	0.10
Neutral	0.20	0.07	77.18	1.01	9.37	0.75	11.91	0.06
Outcome								
Failure	1.41	0.08	75.97	0.99	9.44	0.62	11.94	0.08
Success	1.36	0.08	77.15	1.01	9.40	0.74	11.95	0.07
Valence*Outcome								
Negative Failure	2.62	0.14	75.02	1.01	9.45	0.58	11.99	0.11
Negative Success	2.52	0.13	76.86	1.02	9.48	0.68	11.98	0.10
Neutral Failure	0.21	0.07	76.92	1.02	9.42	0.70	11.89	0.06
Neutral Success	0.19	0.08	77.45	1.05	9.32	0.85	11.92	0.06

#### Test of Hypothesis 1b. Did people use alternative strategies to compensate for ER failure?

We conducted four two-level models with type of affect, manipulated outcome, and their interaction as predictors of self-reported use of distraction, reappraisal, rumination, or none of these strategies. After Bonferroni correction, our critical p-value was 0.0125 (0.05/4 criterion variables).

The effect of manipulated outcome on the self-reported use of distraction did not reach significance based on the above Bonferroni correction, B = 0.18, 95% CI [0.01, 0.35], p = 0.043, nor on the self-reported use of the “none” option, B = −0.30, 95% CI [−0.57, −0.02], p = 0.034. There was also no significant effect of manipulated outcome on the self-reported use of reappraisal, B = 0.11, 95% CI [−0.17, 0.39], p = 0.428, or rumination, B = −0.01, 95% CI [−0.15, 0.14], p = 0.945. Overall, our results do not strongly suggest that participants used alternative ER strategies more following press failure compared to press success. Inferential statistics are reported in Table 3, and descriptive statistics are reported in Table 4.

**Table 3:** Inferential statistics (B, SE, 95% Confidence Intervals, and p-values) from the Two-Level Analyses Examining Effects of Valence, Outcome, and their Interaction on the Use of Compensatory ER Strategies in Study 1.

	Rumination			Distraction			Reappraisal			None		
	Estimate	SE	95% CI	Estimate	SE	95% CI	Estimate	SE	95% CI	Estimate	SE	95% CI
Valence	0.62***	0.13	[0.37, 0.87]	0.34	0.21	[−0.07, 0.75]	0.58**	0.17	[0.24, 0.92]	−1.69***	0.23	[−2.13, −1.24]
Outcome	−0.01	0.08	[−0.15, 0.14]	0.18*	0.09	[0.01, 0.35]	0.11	0.14	[−0.17, 0.39]	−0.30*	0.14	[−0.57, −0.02]
Valence*Outcome	−0.11	0.07	[−0.24, 0.02]	−0.02	0.08	[−0.19, 0.14]	−0.06	0.09	[−0.23, 0.10]	0.03	0.11	[−0.18, 0.23]
Model fit												
AIC	2073.96			1490.04			1341.40			1265.77		
Free parameters	5			5			5			5		
*N*	46			46			46			46		

Note. *p < 0.05, **p < 0.01, ***p < 0.001.

**Table 4:** Parameter Estimates (B and SE) from the Two-Level Analyses Examining Effects of Valence, Outcome, and their Interaction on the Use of Compensatory ER Strategies in Study 1.

	Rumination		Distraction		Reappraisal		None	
	Estimate	SE	Estimate	SE	Estimate	SE	Estimate	SE
Valence								
Negative	0.13	0.21	−1.41	0.21	−1.79	0.20	−3.97	0.52
Neutral	−1.10	0.27	−2.09	0.37	−2.96	0.35	−0.59	0.48
Outcome								
Failure	−0.49	0.21	−1.57	0.23	−2.26	0.30	−2.57	0.49
Success	−0.48	0.23	−1.93	0.23	−2.49	0.24	−1.98	0.45
Valence*Outcome								
Negative Failure	0.02	0.24	−1.26	0.23	−1.74	0.22	−4.23	0.58
Negative Success	0.24	0.22	−1.57	0.24	−1.84	0.24	−3.70	0.55
Neutral Failure	−1.00	0.28	−1.89	0.40	−2.78	0.46	−0.91	0.51
Neutral Success	−1.20	0.31	−2.29	0.38	−3.13	0.35	−0.26	0.48

#### Secondary Analyses. Did people experience more negative emotion after failure on negative compared to neutral trials?

We looked at the interactive effects of type of affect and manipulated outcome in the models described above to answer this question. After Bonferroni correction, our critical p-value was .025 (0.05/2 criterion variables given our focus on self-reported NA and HR). We found no effect of the interaction on self-reported NA, B = 0.02, 95% CI [−0.02, 0.06], p = 0.373, but we did find a significant effect on HR, B = −0.33, 95% CI [−0.59, −0.06], p = 0.015. Inferential statistics are reported in Table 3 and descriptive statistics are reported in Table 4.

#### Did people’s choice of alternative strategies depend on the type of affect in the available situations?

We looked at the interactive effects of type of affect and manipulated outcome in the models described above to answer this question. After Bonferroni correction, our critical p-value was again .0125. We found no effect of the interaction on the self-reported use of distraction, B = −0.02, 95% CI [−0.19, 0.14], p = 0.780, reappraisal, B =−0.06, 95% CI [−0.23, 0.10], p = 0.460, rumination, B =−0.11, 95% CI [−0.24, 0.02], p = 0.095, or the “none” option, B = 0.03, 95% CI [−0.18, 0.23], p = 0.787. Thus, we found no evidence that people’s choice of alternative strategies depends on type of affect in the available situations. Inferential statistics are reported in Table 3, and descriptive statistics are reported in Table 4.

#### Were there individual differences in button-pressing behaviour?

It is notable that 12 (out of 58) participants always pressed the space bar, whereas 13 participants never pressed the space bar. This suggests that there are individual differences in button pressing behaviour. To quantify this variation, we computed the Intraclass Correlation Coefficient (ICC) for button-pressing behaviour to determine the proportion of variance that reliably varies between subjects. Indeed, 70% of variation in button pressing behaviour could be attributed to between subjects differences.

## Discussion

In Study 1, participants ended negative picture presentations more frequently than neutral picture presentations, consistent with the idea that people select situations in light of their emotional potential. However, there was no indication that people used rumination, distraction, or reappraisal in a compensatory way, i.e., more on failure than success trials (no support for Hypothesis 1b). Rather, we found higher HR deceleration on failure relative to success trials (support for Hypothesis 1a), which putatively reflects a difference in emotion experience, but perhaps also an orienting response to the change from picture to black screen only in the success condition^25^. Though interesting, these results are qualified by four primary limitations as follows:

First, by design, whereas the stimuli in the failure condition remained consistently on the screen during the 12-second picture presentation period, the stimuli in the success condition were replaced by a black screen. Any differences between failure and success could, therefore, reflect the presence/absence of stimuli instead of (or in addition to) whether the button press was successful or not.

Second, we did not control whether and when people may have used strategies other than situation selection (the button press) to change their emotions. It could be that situation selection was not the go-to strategy. Participants may, in fact, have tried to use a different ER strategy before they attempted to end the picture presentation, in which case, situation selection via the button press represented compensation for failure of a previously-attempted strategy, which we could not detect.

Third, we used only negative and neutral stimuli. Including positive stimuli could reveal whether people tend to exhibit a hedonic preference or perhaps gravitate towards or away from emotional situations, positive or negative, in this experimental context. In addition, the presence of positive stimuli may make negative stimuli even more upsetting by comparison, thus motivating people to engage in compensatory ER.

Finally, our study sample was fairly homogeneous (Tufts University undergraduates, mostly women, mostly White). It remains uncertain whether our conclusions extend to populations that are more diverse with respect to culture, age, and gender distribution.

## Study 2

The primary goal of Study 2 was to determine whether a sample of community participants would display similar situation selection behaviour by avoiding negative situations, and whether they would use alternative ER strategies to compensate for situation selection failure in an emotional context that includes positive situations. For this effort, we recruited a community sample from a more diverse population by conducting our study online, via Amazon Mechanical Turk. Although we could not use any of the physiological measures from Study 1 in this online context, we retained the self-reported measure of negative affect and added a self-reported measure of positive affect.

To eliminate the inherent difference between the failure and success conditions in presence of the picture stimuli and reduce the likelihood that participants would use strategies other than situation selection first, we adapted our picture task. Specifically, in the first phase of each trial, we presented a picture for 500 ms and prompted participants to indicate whether they wanted to see it again or not. If the participant said they wanted to see the picture again, 70% of the time we would display it again for a few seconds (“yes-success”) and 30% of the time we would display a blank screen instead (“yes-failure”). If the participant said they didn’t want to see the picture again, 70% of the time we would display a blank screen (“no-success”) and 30% we would display the picture again instead (“no-failure”). This allowed us to have success and failure conditions that, averaged across trials, included trials for which there was a change in the information presented from the first to second phase. Thus, both yes-failure and no-success conditions included a shift from picture (500 ms) to no picture (second phase). However, the proportion of trials for which this occurs was different by design (30% yes-failure, 70% no-success).

In addition, we slightly changed the wording (relative to Study 1) when asking participants about alternative ER strategies. We asked: “Which ER strategies did you use after making your choice”? We referred to the time after they made their choice to increase the likelihood that any self-reported ER strategies were used in a compensatory way. In addition, recognizing that participants might have used ER strategies other than the ones we asked them about or none at all, we gave them the option to endorse distraction, reappraisal, rumination, other strategy, or none.

Based on the above changes and our previous work, we formulated three hypotheses. First, we hypothesized that participants would choose situations to different degrees as a function of whether the available situations are pleasant, neutral, or unpleasant. Given that the existing literature provides mixed results in terms of how people select situations as a function of type of affect, we expected to see evidence for one or more of the following patterns: hedonic preference (positive > neutral > negative), contra-hedonic preference (negative > neutral > positive), emotion avoidance preference (neutral > positive and negative), and/or emotion approach preference (positive and negative > neutral). Second, we hypothesized that participants would report using alternative ER strategies more frequently when prevented from receiving the situation of their choice (a failure condition) compared to when receiving the situation of their choice (a success condition). This would constitute evidence for compensation. Lastly, we hypothesized that the compensatory effect may vary as a function of type of affect. For example, participants may use alternative ER strategies most frequently for negative pictures and least frequently for positive pictures. The hypotheses, design, and analysis plan for Study 2 were preregistered prior to data collection through the Open Science Framework, and can be accessed here: http://osf.io/8tgjz.

## Method

### Participants

The study was posted as a ‘Human Intelligence Task’ (HIT) on Amazon Mechanical Turk (MTurk). Participants were paid $10 for agreeing to participate. The HIT was posted daily until we accrued our target sample size of 90 participants with usable data. Participants were at least 18 years old and located in the United States. We also limited the HIT to participation by MTurk workers who have over 97% approval ratings for a minimum of 10,000 HITS in order to maximize data quality.

We excluded data for those participants who did not respond to all trials (N = 0), as well as for those who took longer than 90 minutes to complete the picture task (N = 2). We adopted the latter criterion because long delays might indicate that participants did not complete the task in one sitting, which could introduce unintended variance in the outcomes of interest. In addition, we excluded data for those participants (N = 2) who failed to correctly answer at least 80% of a set of five “catch” trials. On catch trials, instead of a 500-ms picture, participants saw a line of text asking them to simply press a specified number (e.g., 5, or 3, or 2).

Of the 90 participants who provided usable data and passed the data exclusion criteria, 44 were female (48.9%), 75 were White (83.3%), five were multiracial (5.6%), four were African American (4.4%), two were Asian American (2.2%), two were Native American or Alaskan Native (2.2%), and two people did not disclose racial/ethnic information (2.2%). The age range was from 23 to 69 years (M = 36.55 years, SD = 10.03 years). All procedures were approved by the Social, Behavioral, and Educational Research Institutional Review Board at Tufts University and the Army Human Research Protections Office, and all methods were performed in accordance with their guidelines and regulations. Participants provided written informed consent before participating.

### Materials and Procedure

#### Stimuli

Participants viewed a set of 126 digital colour pictures (800 pixels × 600 pixels) selected from the IAPS^19^. We established three comparable sets of 42 stimuli: positive, neutral, and negative pictures. The positive pictures were highly pleasant (M = 7.23, SD = 0.46, on a scale ranging from 1 to 9, where 9 = completely happy), and highly arousing (M = 6.20, SD = 0.53, on a scale ranging from 1 to 9, where 9 = completely aroused). The neutral pictures were neither pleasant nor unpleasant (M = 5.18, SD = 0.46), and low in arousal (M = 3.13, SD = 0.29). The negative pictures were highly unpleasant (M = 2.38, SD = 0.51), and highly arousing (M = 6.21, SD = 0.47). The catalogue numbers of pictures from the IAPS 2008 set used in this study can be found in Supplementary Information.

#### Picture task

Participants provided informed consent via Qualtrics, where they were subsequently asked to rate their experience of various emotions at the moment. Then they were directed to Inquisit 4.0 (Millisecond.com), which we used to program and present the picture task. Participants viewed the above set of 126 pictures, evenly distributed across six blocks. The order in which the 126 pictures were presented was randomized for each participant.

**Figure 2:**
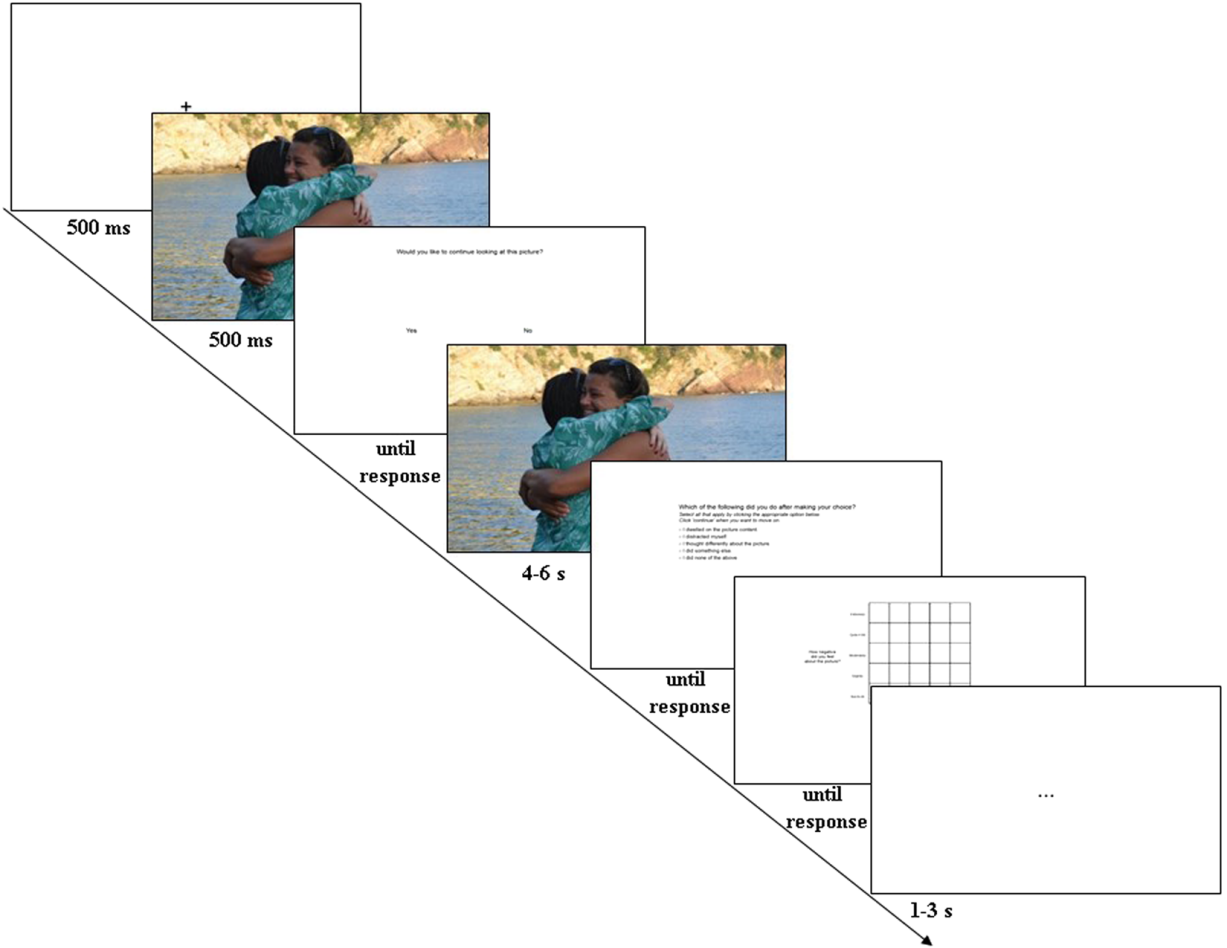
Trial structure for the picture task in Study 2. Whereas we used IAPS pictures for this task, the example picture in this figure is from one author’s private collection.

All trials began with a white fixation cross presented in the centre of a black screen for 1 s. The fixation was followed by the presentation of a picture for 500 ms. Participants were then asked whether they wanted to see the picture again (yes or no). This picture presentation time was long enough for participants to decide whether they wanted to see this picture again, while being too short to allow them to launch other forms of ER. If participants indicated they wanted to see it again, the picture either: (1) reappeared for between 4–8 s (“yes-success” condition; approximately 70% of the press trials) or (2) did not reappear, and participants saw a blank screen for 4–8 s (“yes-failure” condition; approximately 30% of the press trials). If participants indicated they did not want to see it again, the picture either: (1) did not reappear, and participants saw a blank screen for 4–8 s (“no-success” condition; approximately 70% of the press trials) or (2) reappeared for between 4–8 s (“no-failure” condition; approximately 30% of the press trials) (see Fig. 2). Post situation selection picture presentation time (approximately 6 s average) is consistent with previous work using similar paradigms^26,27^.

After picture presentation, participants responded to this question on screen: “Which of the following did you do after making your choice? Select all that apply by clicking the appropriate option below: (1) I dwelled on the picture content; (2) I distracted myself; (3) I thought differently about the picture; (4) I did something else; (5) I did none of the above”. They could endorse more than one option. Option 1 reflected rumination (thought about the content of the picture and how it made them feel), option 2 reflected distraction (thought about something other than the picture), option 3 reflected reappraisal (thought differently about/reinterpreted the picture), option 4 reflected engaging in an ER strategy other than the first three options, and option 5 reflected using no ER strategies.

During the training procedure for the picture task, participants learned what the strategies listed as options meant, and were given examples of ways in which these strategies might be used, e.g., for “I dwelled on the picture content” an example was: “kept thinking about the picture”; they also had nine practice trials, to make sure they understood the task and the distinctions between these different strategies (see Supplementary Information). The ER strategy screen remained present until participants provided a response.

After reporting which ER strategies they used, participants provided a rating of negative and positive affect using a variant of the evaluative space grid (ESG)^20^, allowing us to efficiently collect two independent ratings. Participants chose one number in the 5x5 grid representing their ratings of how positive they felt during the picture trial (on the X axis) and how negative they felt during the picture trial (on the Y axis). The rating screen remained present until a response was recorded. Lastly, participants saw a blank screen with a central black ellipsis that lasted between 1 and 3 s (randomly determined) to provide a brief break between trials.

At the end of the picture task, participants completed a number of measures administered as possible exploratory moderators of performance. First, their internet browser automatically directed them to an online measure of acute and chronic stressors, the Stress and Adversity Inventory^28^. Upon completion of the survey, participants’ browsers were directed to a Qualtrics survey in which we asked about participants’ thoughts, feelings, and reactions during the task, and their experience of various emotions at the moment. In addition, we administered questionnaires asking about grit (Short Grit Scale^29^), cognitive flexibility (Cognitive Flexibility Scale^30^), and self-control (Brief Self Control Scale^31^). Lastly, we obtained demographic information (gender, age, race, etc.). (Note that we do not include exploratory moderation analyses as part of this manuscript.) After completing this survey, participants were debriefed, thanked for their participation, and dismissed. We issued compensation thereafter.

### Independent Variables

We manipulated two independent variables in our picture task. One was the type of affect elicited by the pictures with three conditions (high-arousal negative, low-arousal neutral, or high-arousal positive). The other was the manipulated outcome of their decision to see or not see the picture again; we collapsed across “yes” and “no” decisions to examine two conditions (failure or success).

### Dependent Variables

To assess situation selection, trials on which participants clicked ‘yes’ to see the picture again were coded as 1, and trials on which participants clicked ‘no’ were coded as 0. Ratings of negative and positive affect ranged from 1 to 5 (‘not at all’ to ‘extremely’).

There were five variables reflecting the alternative ER strategies participants could have endorsed: distraction, reappraisal, rumination, other, or none (1 = endorsing the strategy, 0 = not endorsing). In addition, at the trial level, we combined the reported use of the four alternative ER strategies (distraction, reappraisal, rumination, other) into a single score to indicate whether any alternative ER strategies were used. This was a categorical variable, with the value of 1 indicating that one or more alternative ER strategies were used on the particular trial, and the value of 0 indicating that no alternative ER strategies were used on the particular trial. We also had a variable indicating the number of strategies used on each trial (scores could range from 0 to 4, including the “other” strategy option).

### Data Analysis and Retention

Multilevel analyses with two levels were conducted using full information maximum likelihood estimation with robust standard errors in Mplus v. 7.11^24^. Continuous predictors were grand-mean centred. For analyses with continuous criterion variables, we estimated predictors as random effects that could thus vary between subjects. However, unless otherwise noted, for analyses with categorical criterion variables, we estimated predictors as fixed effects because the models could not converge otherwise. We used Bonferroni correction to determine significance levels based on the number of comparisons conducted for each hypothesis test. All 90 participants provided data for all analyses, so there were no exclusions.

## Results

### Summary of Preliminary Analyses

Preliminary analyses revealed that our stimuli had the desired effect. Namely, participants reported experiencing more negative affect on negative compared to both neutral and positive trials. They also reported experiencing more positive affect on positive compared to both neutral and negative trials. Details of the statistical analyses are provided under Supplementary Information.

### Hypothesis Testing

#### Test of Hypothesis 1. Was people’s use of situation selection as an ER strategy a function of whether the available situations were positive, neutral, or negative?

We conducted a two-level model testing the main effect of type of affect on within-subjects, trial-by-trial variation in use of situation selection as an ER strategy, analysed as a categorical criterion variable. We estimated predictors as random effects that could thus randomly vary between subjects, since the model converged successfully. Type of affect was represented by two contrasts: positive vs. negative situations (positive [1], neutral [0], negative [−1]) and emotional vs. neutral situations (positive [1], neutral [−2], negative [1]). Given that we used only one dependent variable to test this hypothesis, no Bonferroni correction was applied.

Supporting Hypothesis 1, there was a significant effect of both affect contrasts, positive versus negative pictures B = 1.95, 95% CI [1.65, 2.25], p < 0.001, and emotional versus neutral B = −0.18, 95% CI [−0.30, −0.06], p = 0.004. Additional model constraints indicated that participants preferred positive over neutral pictures, B = 1.42, 95% CI [1.08, 1.77], p < 0.001, and neutral over negative pictures, B = −2.48, 95% CI [−3.04, −1.92], p < 0.001. The odds ratio of selecting positive situations was 20.23, compared to only 4.88 for neutral, and 0.41 for negative situations. Overall, this pattern supports a hedonic preference with respect to situation selection.

#### Test of Hypothesis 2. Did people use alternative ER strategies to compensate for ER failure?

We conducted two two-level models with the two affect contrasts, manipulated outcome, and their interaction as predictors of the use of alternative ER strategies. The contrast used to represent manipulated outcome was failure (1) vs. success (−1). After Bonferroni correction, the p-value for these two models was 0.025 (0.05/2 criterion variables). Inferential statistics for all analyses are presented in Table 5, and descriptive statistics are presented in Table 6.

**Table 5:** Statistics (B, 95% Confidence Intervals, and p-values) from the Two-Level Analyses Examining Effects of Valence, Outcome, and their Interaction on the Use of Compensatory ER Strategies in Study 2.

	Number strat. used			0 vs. at least 1 strat. used			Rumination			Distraction			Reappraisal			Other		
	Estimate	SE	95% CI	Estimate	SE	95% CI	Estimate	SE	95% CI	Estimate	SE	95% CI	Estimate	SE	95% CI	Estimate	SE	95% CI
Valence																		
Posneg	−0.08***	0.01	[−0.11, −0.05]	−0.57***	0.14	[−0.85, −0.29]	0.42***	0.09	[0.25, 0.60]	−1.18***	0.12	[−1.41, −0.94]	−0.35***	0.08	[−0.51, −0.19]	0.04	0.11	[−0.18, 0.25]
Emoneu	0.05***	0.01	[0.04, 0.07]	0.46***	0.06	[0.34, 0.58]	0.18***	0.04	[0.09, 0.27]	0.20***	0.05	[0.10, 0.30]	0.03	0.04	[−0.05, 0.11]	−0.16*	0.07	[−0.30, −0.02]
Outcome	−0.02	0.01	[−0.03, 0.00]	−0.22*	0.1	[−0.42, −0.03]	−0.22***	0.06	[−0.33, −0.11]	0.06	0.07	[−0.08, 0.20]	−0.03	0.06	[−0.14, 0.07]	0.31***	0.08	[0.16, 0.46]
Valence*Outcome																		
Posxout	−0.02**	0.01	[−0.04, −0.01]	−0.19*	0.1	[−0.38, −0.00]	−0.19***	0.05	[−0.29, −0.10]	0.09	0.07	[−0.04, 0.22]	−0.07	0.04	[−0.16, 0.02]	0.05	0.07	[−0.08, 0.18]
Emoxout	0.01*	0	[0.00, 0.02]	0.04	0.04	[−0.03, 0.11]	0.05*	0.02	[0.01, 0.10]	−0.05	0.04	[−0.12, 0.03]	0	0.03	[−0.06, 0.07]	0.05	0.03	[−0.02, 0.12]
Model fit																		
AIC	10846.42			5790.90			10950.89			6825.64			6904.26			4342.99		
Free parameters	13			7			7			7			7			7		
*N*	90			90			90			90			90			90		

Note. *p < 0.05, **p < 0.01, ***p < 0.001. posneg = valence contrast (positive [1], neutral [0], negative [−1]), emoneu = valence contrast (positive [1], neutral [−2], negative[1]); posxout = interaction between “posneg” valence contrast (positive[1], neutral [0], negative [−1]) and manipulated outcome (failure [1] vs. success [−1]); emoxout = interaction between “emoneu” valence contrast (positive [1], neutral [−1], negative [1]) and manipulated outcome (failure [1] vs. success [−1]).

**Table 6:** Parameter Estimates (B and SE) from the Two-Level Analyses Examining Effects of Valence, Outcome, and their Interaction on the Use of Compensatory ER Strategies in Study 2.

	Number strat. used		0 vs. at least 1 strat. used		Rumination		Distraction		Reappraisal		Other	
	Estimate	SE	Estimate	SE	Estimate	SE	Estimate	SE	Estimate	SE	Estimate	SE
Valence												
Positive	0.91	0.03	−3.49	0.37	0.00	0.20	1.78	0.19	2.43	0.21	3.81	0.29
Neutral	0.83	0.04	−4.30	0.39	−0.96	0.21	2.35	0.20	2.69	0.22	4.25	0.32
Negative	1.07	0.04	−2.36	0.39	−0.84	0.26	4.13	0.30	3.13	0.25	3.74	0.32
Outcome												
Failure	0.92	0.04	−3.61	0.37	−0.82	0.21	2.81	0.21	2.72	0.21	4.24	0.29
Success	0.95	0.03	−3.16	0.36	−0.38	0.21	2.69	0.21	2.79	0.22	3.62	0.29
Valence*Outcome												
Positive Failure	0.88	0.04	2.90	0.38	0.84	0.24	−3.61	0.28	−3.17	0.25	−3.65	0.32
Positive Success	0.94	0.03	3.65	0.39	1.56	0.24	−3.83	0.29	−2.97	0.24	−4.46	0.32
Neutral Failure	0.80	0.04	2.17	0.38	−0.08	0.24	−3.00	0.27	−2.85	0.26	−3.41	0.32
Neutral Success	0.87	0.04	2.77	0.37	0.57	0.24	−3.31	0.27	−2.77	0.24	−3.82	0.32
Negative Failure	1.08	0.04	4.42	0.43	0.38	0.22	−1.45	0.20	−2.33	0.23	−3.81	0.34
Negative Success	1.05	0.04	4.41	0.48	0.33	0.22	−1.30	0.20	−2.41	0.22	−4.44	0.35

Note. All values represent log odds, except for “number of strategies used” which represent mean values.

#### Use of ER strategies in general

In the first model, we had a continuous dependent variable indicating how many alternative ER strategies people reported using, ranging on a scale from zero to four strategies. Both affect contrasts, outcome, and their interactions were estimated as random effects. This model showed no significant effect of manipulated outcome, B = −0.02, 95% CI [−0.03, 0.00], p = 0.066.

In the second model, we examined the categorical dependent variable, indicating whether people used no alternative ER strategies (0) or at least one alternative strategy (1). Both affect contrasts, outcome, and their interactions were estimated as fixed effects. This model revealed that people reported using at least one ER strategy compared to using no strategies at all on success compared to failure trials, B = −0.22, 95% CI [−0.42, −0.03], p = 0.024, the opposite of the predicted direction. Overall, these results do not support Hypothesis 2; there is no evidence that participants used alternative strategies to compensate for situation selection failure.

#### Use of specific ER strategies

To determine which specific strategies participants endorsed, we conducted similar models as described above, but with the use of rumination, distraction, reappraisal, or other ER strategies as single dependent variables. Both affect contrasts, outcome contrasts, and their interactions were estimated as fixed effects. After Bonferroni correction, the p-level for these analyses was 0.0125(0.05/4 criterion variables).

Contrary to our prediction, people reported using rumination more frequently when the outcome of situation selection was successful, compared to when it failed, B = −0.22, 95% CI [−0.33, −0.11], p < 0.001. In addition, there was no significant effect of manipulated outcome on the use of distraction, B = 0.06, 95% CI [−0.08, 0.20], p = 0.376, or reappraisal, B = −0.03, 95% CI [−0.14, 0.07], p = 0.543. Lastly, participants reported using “other” ER strategies more frequently on failure compared to success trials, B = 0.31, 95% CI [0.16, 0.46], p < 0.001. Inferential statistics for all analyses are presented in Table 5, whereas mean values and standard errors are presented in Table 6.

#### Test of Hypothesis 3. Did people’s choice of alternative strategies depend on the type of affect elicited in the available situations?

We looked at the previously conducted models, focusing on the interactions of the two affect contrasts with manipulated outcome on the use of alternative ER strategies. As will be evident, overall, these results do not provide evidence of an impact of type of affect on compensation for situation selection failure using alternative ER strategies.

#### Use of ER strategies in general

**Figure 3:**
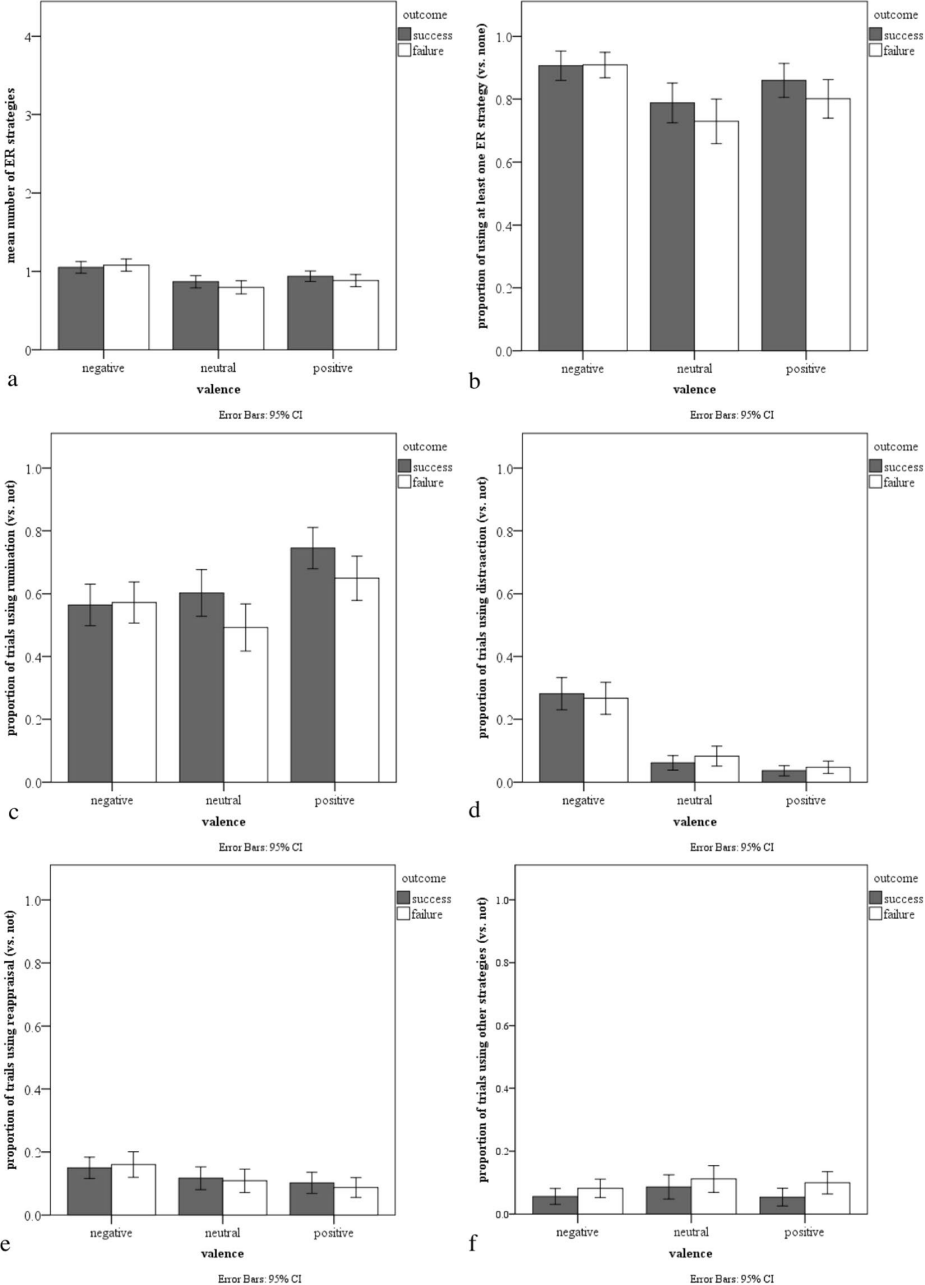
(**a**) Estimated mean number of alternative ER strategies (and 95% CI) on failure and success trials, for positive, neutral, and negative pictures in Study 2. Proportion of Study 2 trials (and 95% CI) on which participants reported at least one ER strategy compared to no ER strategies at all (**b**), rumination (**c**), distraction (**d**), reappraisal (**e**), or other strategies (**f**) on failure and success trials, for positive, neutral, and negative pictures.

After Bonferroni correction, the p-value for the analyses in this subsection was 0.025 (0.05/2 criterion variables). When using the number of ER strategies as the dependent variable, both interaction terms showed significant effects: positive vs negative x manipulated outcome, B = −0.02, 95% CI [−0.04, −0.01], p = 0.004, and emotional vs neutral x manipulated outcome, B = 0.01, 95% CI [0.00, 0.02], p = 0.023. Model constraints in which we evaluated the effect of failure [1] versus success [−1] within each affect category revealed that, on both positive and neutral trials, participants reported using more strategies on success compared to failure trials, B = −0.06, 95% CI [−0.10, −0.01], p = 0.024, and, B = −0.07, 95% CI [−0.12, −0.02], p = 0.009, respectively. However, there was no significant difference between failure and success on negative trials, B = 0.03, 95% CI [−0.01, 0.07], p = 0.155 (see Fig. 3, chart a). Inferential statistics for this analysis are presented in Table 5, and descriptive statistics can be found in Table 6.

When using the categorical dependent variable indicating whether people used no alternative ER strategies (0) or at least one alternative strategy (1), the model revealed no significant interaction between positive vs. negative x manipulated outcome, B = −0.19, 95% CI [−0.38, 0], p =0.048, and no significant interaction between emotional vs. neutral x manipulated outcome, B = 0.04, 95% CI [−0.03, 0.11], p = 0.278 (see Fig. 3, chart b). Inferential statistics for this analysis are presented in Table 5, and descriptive statistics can be found in Table 6.

#### Use of specific ER strategies

After Bonferroni correction, the p-value for the analyses in this subsection was 0.0125 (0.05/4 criterion variables). In terms of specific strategies, the interaction between positive vs. negative x manipulated outcome had a significant effect on the use of rumination: B = −0.19, 95% CI [−0.29, −0.10], p < 0.001; the emotional vs neutral x manipulated outcome interaction was not significant, B = 0.05, 95% CI [0.01, 0.10], p = 0.016. Follow-up model constraints as above revealed that, on both positive and neutral trials, participants reported using rumination more frequently on success compared to failure trials, B = −0.71, 95% CI [−1.05, −0.38], p < 0.001, and B = −0.65, 95% CI [−0.93, −0.37], p < 0.001, respectively. No significant difference between failure and success was revealed on negative trials, B = 0.06, 95% CI [−0.21, 0.33], p = 0.676 (see Fig. 3, chart c). Neither interaction term had a significant impact on the use of distraction, reappraisal, or “other” (see Fig. 3d–f, and Table 5 for inferential statistics).

#### Secondary Analysis. Were there individual differences in choosing whether to see the pictures again?

As in Study 1, we computed ICC to determine to what extent there are individual differences, this time in saying yes or no to seeing the picture again. In Study 2, 30% of variation in saying yes or no to seeing the picture again could be attributed to between subject differences. A total of 8 (out of 90) participants always wanted to see the pictures again, whereas only 1 participant never wanted to see the pictures again. Looking at specific affect categories, 27 participants always wanted to see positive pictures again, compared to 19 and 10 who always wanted to see neutral and negative picture again, respectively. Only 3 participants never wanted to see positive pictures again, compared to 6 and 10 participants who never wanted to see neutral and negative pictures again, respectively. Individual differences thus were prominent, albeit less so than in Study 1.

## Discussion

In Study 2, our participants displayed an overwhelming hedonic preference for positive over neutral and neutral over negative situations (support for Hypothesis 1). However, we did not find robust evidence for ER compensation in light of situation selection failure (little support for Hypotheses 2 and 3). The only finding that suggested support for the use of compensatory ER after situation selection failure was the observation that strategies other than rumination, distraction, and reappraisal (i.e., selecting “other”) were used more frequently on failure vs. success trials. Otherwise, the people we studied did not report using a greater number of ER strategies in the failure compared to the success condition, nor were they more likely to use any alternative ER strategy or to specifically use distraction and reappraisal more in those circumstances. Instead, they reported using rumination more frequently on success compared to failure trials in the positive and neutral (but not negative) conditions. Although this could indicate that participants used rumination to amplify their experience of pleasant (or at least not unpleasant) stimuli and that failure of situation selection derailed this strategy, this pattern does not lend clear support to the compensatory ER idea.

Although we believe the design of Study 2 provided us with better opportunities to capture situation selection and ER compensation compared to Study 1, there are some limitations that need to be considered. Whereas we purposefully administered the study online in order to reach a more diverse sample, the settings in which the participants completed the task were not fully controlled. We asked the participants to complete the study in one sitting in a quiet place, but we had no real control beyond excluding the data of anyone who took longer than 90 minutes or missed more than 80% of catch trials. In addition, we were not able to collect any physiological signals, which meant we were not able to determine whether there were any bodily changes associated with situation selection failure as in Study 1, in which we observed HR deceleration. Lastly, because our participants completed the task online, we did not have the opportunity to address any questions they might have had. It is possible that participants interpreted the ER strategies differently than intended.

## General Discussion

Using picture-based laboratory tasks, the overall goals of this research were to assess to what extent people would choose situations based on the affective nature of those situations, and whether people engage in compensatory ER when their attempts at situation selection fail. Consistent with the idea that people choose situations based on affective potential, both studies showed that people preferred to see neutral over negative pictures. Study 2 further showed that people preferred positive pictures over neutral ones.

Despite clear evidence of situation selection reflecting hedonic preference, we found very little support for the idea that participants would use alternative ER strategies in the wake of situation selection failure. In Study 1, there was no indication that people used any of the alternative ER strategies we assessed in a compensatory way, i.e., more on failure than success trials. In Study 2, participants reported using strategies other than rumination, distraction, and reappraisal more frequently on failure compared to success trials, consistent with a compensatory interpretation. However, there were no such effects on the number of alternative strategies, the use of any alternative strategies at all, or on specific use of distraction or reappraisal, which we would have expected to see. Moreover, although participants did endorse ruminating on success trials more than failure trials in the positive and neutral (but not negative) conditions, it is not clear that this should be interpreted as compensatory ER.

Why did we generally fail to reveal evidence that people use ER strategies in a compensatory way, as theorized in the SOC-ER framework? One obvious answer could be that the theory is wrong and they don’t. However, it is also possible that one or more aspects of our method were simply not sensitive to detecting it. Having to endure no more than 6–12 seconds of an undesirable situation may be insufficient to motivate identifying and using alternative ER strategies. It’s also possible that there simply isn’t enough time to do so or that doing so is a skill that requires practice over more situations than we studied herein.

Although we did not find strong evidence to support the compensation piece of the SOC-ER framework, previous studies are consistent with the idea that different strategies have different affective, cognitive, and social consequences and are not all equally suited for all circumstances^32^. Indeed, research showed that people used multiple ER strategies in response to a disgust-eliciting film clip^33^, and whereas distraction was effective when initiated late during a sadness-evoking film, reappraisal was not effective when initiated late^34^. Thus, different strategies may be differently effective at various times during an emotion-eliciting event. Lastly, research shows that people use ER flexibly, depending on their individual characteristics and the context of regulation^35,36^. Notably, our present work contributes to the growing body of literature on the extent to which people choose situations in accordance with their affective preferences. In these two studies, we found that people display a hedonic preference when choosing situations. This finding is consistent with the hedonic principle^37^ and is present in some of the previous work on situation selection^11^. However, previous work has also produced evidence for situation selection that reflects a contra-hedonic preference^8^ or found no difference in situation selection based on different valence categories^9,10,12,38^. The presence of hedonic and contra-hedonic situation selection across studies could be driven by factors such as participants’ age^11^, attitude towards emotions^39^, their emotional goals^40–43^, or motivation^44^, social context of ER^40^, as well as levels of arousal^9^, interest^9,45^, or attractiveness of the stimuli^46^. Why we observed the hedonic pattern in this particular context is unclear; efforts to understand the mixed nature of findings in this domain deserve attention in future studies.

As noted above, one potential factor that may explain variability across studies in hedonic and contra-hedonic situation selection could be the extent to which stimuli/situations vary in levels of arousal. We chose our negative and positive stimuli to be highly unpleasant and pleasant, respectively, but also high in arousal. We chose our neutral stimuli, by contrast, to be neither unpleasant nor pleasant, and low in arousal. Thus, our manipulation of affective categories, which we’ve labeled negative, neutral, and positive in this work, purposely confounds valence and arousal. We designed our study as such based on our previous work^9^, which showed that such stimuli prompted situation selection most frequently. In that study, participants most frequently said they terminated presentation of high-arousal negative pictures because they were upset and low-arousal neutral pictures because they were bored. Future work could benefit from orthogonally manipulating valence and arousal so that these dimensions can be treated as independent predictors of people’s choice of situations.

Selecting situations based on how they can make us feel deserves further study because this process has consequences for our well-being. When feeling upset, people tend to prioritize short-term ER goals over other goals, so they may indulge in eating unhealthy snacks and procrastination^47^. On the other hand, whereas people generally seek out pleasure, they are also willing to experience unpleasant emotions if that will help their long-term goals^43^. Those individuals who frequently choose situations that bring short-term pleasure, such as eating food high in carbohydrates and fat, consuming alcohol, and substance abuse, can develop problems with obesity and addiction. Understanding the mechanism through which we select situations can help us give recommendations and guidelines for prevention of obesity and addiction.

The use of situation selection can play an important role in mental health, too. One common example of situation selection is experiential avoidance, defined as a person’s unwillingness to experience negative emotions, sensations, feelings and thoughts, and desire to change the form or frequency of situations giving rise to those experiences^48^. Unhealthy avoidance of emotions is implicated in many behavioural disorders such as substance abuse and dependence, obsessive compulsive disorder, panic disorder with agoraphobia, and borderline personality disorder, and suicide as the ultimate avoidance strategy^48^. Thus, studying which situations people choose to approach, avoid, or modify, how frequently, in which contexts, and the cognitive, emotional, and social consequences of doing so can inform clinical practice and help in the development of appropriate therapy. The present set of studies is a step towards meeting those goals.

Though strong in many respects, there are reasons to question the ecological validity of these studies. For one, our participants received an explicit opportunity to end the situation (Study 1) or indicate whether they wanted to be in the situation again (Study 2); in everyday life, people are rarely offered such explicit, concrete opportunities to regulate their emotions. In addition, it is likely that people’s short-term emotional goals (in the lab) differ from their long-term emotional goals (in the real word), thus leading them to choose situations differently. In fact, there is evidence that people’s ER is guided by their social context, motivation, and regulatory goals. Supporting the notion that situation selection may operate differently in everyday life, studies in which people were asked to report about their real-life choices of situations found no relationship between the percentage of time spent in certain situations and the affect experienced in those situations^38^, or a difference in the frequency with which people choose positive (e.g. listening to an upbeat song) or negative situations (e.g. watching a horror film)^8^. Future work should expose participants to situations in the lab that are closer to real-life experiences, or should use methods like experience sampling or journaling in the real world. Such efforts have promise in yielding better estimates of the use of situation selection in daily life.

Lastly, we uncovered substantial between-subjects variation in situation selection in our picture tasks. It is, therefore, important to consider additional between-subjects predictors in future studies, some of which may moderate the effects we have observed in this set of studies. For instance, age is one of the factors that could be related to how people regulate their emotions^14^, in particular how they use situation selection and modification strategies^11,12^. There is also evidence that women use most ER strategies more frequently^49^ and differently^50^ than men. In terms of situation selection strategies, some studies found that women were more likely to use avoidant coping strategies^51^ and more likely to avoid distressing situations^49^, whereas other studies found that men report using avoidant strategies more than women^52^. In addition, personality traits and abilities like self-control and cognitive flexibility are also linked to ER. Regulating negative affect requires self-control^53^, and cognitive flexibility predicts one’s ability to regulate emotions^54^. In particular, people lower in trait self-control are more likely to engage in avoidant coping^55^, and cognitive flexibility is related to approach/avoidance behaviour^56^, suggesting that both of these may play a role in how and when people use situation selection. Finally, stress and adversity could play a role in ER– following an acute stress induction in lab, people were not able to effectively use cognitive ER to change how they feel^57^. Severity of PTSD symptoms has also been linked to difficulties in ER^58^. Avoidance may alleviate stress and anxiety, whereas approach can provide us more control over the stressor^59^. Considering all of the above, it would be useful to determine whether the situations people approach and avoid based on affect vary as a function of relevant individual differences, as well as whether compensation for ER failure emerges when taking one or more of those individual differences into account.

In sum, the goals of these two studies were to: (1) determine the extent to which people choose situations as a function of their emotional potential in the laboratory, and (2) test the notion of compensatory ER as proposed in the SOC-ER framework. To accomplish these goals, in Study 1, we used a picture-based task similar to our previous work^9^ in which participants had the option of pressing a button to end negative and neutral situations. In Study 2, we developed an online task in which, after viewing positive, neutral, and negative pictures for 500 ms each, the participants indicated whether they wanted to see the picture again. We manipulated the outcome of situation selection in both studies such that the outcome was or was not congruent with participants’ wishes. The results of Study 1 revealed a tendency of people to stay in neutral situations more than in negative ones, but provided no evidence for ER compensation following failure; rather, participants seemed to experience more negative affect in the failure compared to the success condition, as reflected in HR deceleration. Study 2 revealed that participants have a hedonic preference for situations, insofar as they chose to see positive pictures again more frequently than neutral pictures, and neutral more frequently than negative. Study 2 also, however, revealed limited evidence of use of compensatory strategies; participants reported using strategies “other” than rumination, distraction, and reappraisal more frequently following failure but other comparisons of a priori interest failed to support the compensatory ER hypothesis. Overall, we conclude that, although people choose situations based on affect (i.e., attempt to end or avoid negative situations and pursue pleasant ones), they do not use the alternative ER strategies that we assessed to compensate for situation selection failure in this experimental laboratory context.

## Electronic supplementary material

Supplementary Information
