# Supplementary material for: Emotion Regulation Compensation Following Situation Selection Failure
Source: Sci Rep. 2018 Apr 3;8:5411. doi: 10.1038/s41598-018-23654-2 (PMC5882914; doi:10.1038/s41598-018-23654-2)
Supplement: Supplementary file 1 — Supplementary Information [file 41598_2018_23654_MOESM1_ESM.pdf]

Emotion Regulation Compensation Following Situation Selection Failure

Lara Vujovic

Heather L. Urry

Supplementary Information

Supplementary Study Materials

Study 1 stimuli

The following pictures from the International Affective Picture System 2008 set, listed by catalogue number, were used in Study 1: negative high arousal – 2683, 2811, 3000, 3001, 3010, 3016, 3053, 3060, 3069, 3080, 3120, 3130, 3170, 3195, 3212, 3266, 3500, 3530, 6250.1, 6313, 6315, 6350, 6510, 6540, 6550, 6563, 6570.1, 8230, 8485, 9075, 9252, 9254, 9410, and 9413; neutral low arousal – 2020, 2026, 2036, 2038, 2102, 2104, 2190, 2200, 2210, 2221, 2235, 2357, 2381, 2383, 2393, 2396, 2397, 2411, 2440, 2480, 2493, 2495, 2499, 2513, 2570, 2620, 2745.1, 2840, 2850, 2870, 2890, 5410, 5875, and 7493.

Study 1 practice trials instructions

Participants saw a picture of a man aiming a polar bear with a gun. On three different screens, they read the instructions for each of the three offered strategies:

1. "Dwelled on the picture content" (option 1) could involve one or more of the following:

- kept thinking about the picture even after it went away
- kept seeing the bear and/or the shooter in my mind's eye
- couldn't stop thinking about the injured bear
- kept imagining the fear and the pain the bear experienced
- thought back to the shooter and his thoughts and feelings in this situation

2. "Distracted myself with other thoughts" (option 2) could involve one or more of the following:

- looked at the snow/sky instead of the bear/shooter
- thought about your grocery list
- thought about a movie you saw recently
- thought about something that happened to you on the way here
- thought about your friend's birthday party from three years ago

3. "Thought differently about the picture" (option 3) could involve one or more of the following:

- thought the shooter was aiming at something behind the bear
- thought the shooter missed and the bear was OK in the end
- thought the shooter was starving and the bear was his only available food
- thought the bear had to be put down due to illness

- thought the picture was staged

Study 2 stimuli

The following pictures from the International Affective Picture System 2008 set, listed by catalog number, were used in Study 2: negative – 2095, 2683, 2703, 2800, 2811, 3180, 3230, 3350, 3550, 6211, 6212, 6220, 6250, 6312, 6315, 6350, 6370, 6520, 6550, 6560, 6563, 6570.1, 6821, 6834, 6840, 8230, 8480, 8485, 9050, 9075, 9163, 9250, 9254, 9332, 9400, 9413, 9414, 9425, 9800, 9810, 9905, and 9910; neutral – 2020, 2026, 2036, 2038, 2102, 2104, 2190, 2200, 2210, 2214, 2215, 2221, 2235, 2357, 2377, 2381, 2383, 2384, 2393, 2396, 2397, 2411, 2440, 2480, 2493, 2495, 2499, 2506, 2512, 2513, 2518, 2570, 2593, 2620, 2745.1, 2840, 2850, 2870, 2890, 5410, 5875, and 7493; positive – 2045, 2216, 2303, 2347, 2389, 4597, 4599, 4626, 4640, 5460, 5470, 5621, 5629, 7502, 7660, 8021, 8030, 8034, 8080, 8090, 8116, 8158, 8161, 8163, 8179, 8180, 8185, 8186, 8190, 8193, 8200, 8206, 8210, 8300, 8370, 8400, 8420, 8470, 8490, 8492, 8496, and 8499.

Study 2 practice trials instructions

Participants saw a picture of a man aiming a polar bear with a gun. On four different screens, they read the instructions for each of the four offered strategies:

1. "I dwelled on the picture content": Thinking about the picture of the man and the bear you can see below, you would select ‘I dwelled on the picture content’ if you did one or more of the following:

- kept thinking about the picture
- kept seeing the bear and/or the shooter in my mind's eye
- couldn't stop thinking about the injured bear
- kept imagining the fear and the pain the bear experienced
- thought back to the shooter and his thoughts and feelings in this situation

2. "I distracted myself with other thoughts": Thinking about the picture of the man and the bear you can see below, you would select ‘I distracted myself with other thoughts’ if you did one or more of the following:

- looked at the snow/sky instead of the bear/shooter
- thought about your grocery list
- thought about a movie you saw recently
- looked away from the picture
- closed your eyes

3. "I thought differently about the picture”: Thinking about the picture of the man and the bear you can see below, you would select ‘I thought differently about the picture’ if you did one or more of the following:

- thought the shooter was aiming at something behind the bear
- thought the shooter missed and the bear was OK in the end
- thought the shooter was starving and the bear was his only available food
- thought the bear had to be put down due to illness
- thought the picture was staged

4. "I did something else”: Thinking about the picture of the man and the bear you can see below, you would select ‘I did something else’ if you did one or more of the following or anything else to change how you feel:

- took a deep breath
- kept your face still
- felt for the bear and/or shooter
- tried to better understand the situation depicted

Supplementary Results

Study 1 Preliminary Analyses

Did people respond with more negative emotion to negative than neutral pictures? To answer this question, we conducted four two-level models testing the main effect of valence (negative [1] vs. neutral [-1]) on within-subjects (trial-by-trial) variation in emotional responding (as reflected in negative affect (NA) rated at the end of each trial, HR, corrugator activity, and EDA prior to button press). To isolate variation as a function of picture onset, mean activity during the pre-button press reactivity period was regressed on baseline activity (mean of activity occurring 1 s prior to picture onset) for each of the three physiological measures. We focused only on pre-button press reactivity to be able to establish whether heightened emotional response lead to the button press. The valence contrast was estimated as a random effect that could, thus, vary between subjects. After applying a Bonferroni correction, our critical p-value changed to .0125.

As expected, participants reported experiencing more NA on negative compared to neutral trials, B = 1.10, 95% CI [1.01, 1.19], $p < .001$, N == 58. In addition, HR was slower on negative compared to neutral trials, B = -0.78, 95% CI [-1.27, -0.29], $p = .002$, N == 42. However, there was no difference in corrugator activity between negative and neutral trials, B = 0.16, 95% CI [-0.70, 1.02], $p = .719$, N == 44. There was also no difference in EDA between negative and neutral trials prior to button press, B = 0.01, 95% CI [-0.02, 0.03], $p = .478$, N == 43. Overall, participants' subjective experience and the change in HR belied more negative emotion on negative than neutral trials; our picture set was, thus, successful in inducing the target emotional states.

Were people more motivated to regulate their emotions on negative than neutral trials? To answer this question, we conducted a two-level model in which self-reported motivation to change emotion was regressed on the valence contrast (estimated as random effect). No Bonferroni correction was applied because there was only one dependent variable in this analysis. Results revealed that people were significantly more motivated to regulate their emotions on negative trials relative to neutral trials, B = 0.76, 95% CI [0.64, 0.87], $p < 0.001$, N == 58.

Did people use situation-targeted ER more on negative than neutral trials and were they faster to do so? We conducted a two-level model in which button pressing behavior (whether people pressed the space bar or not on a given trial) was regressed on the valence contrast (negative [1] vs. neutral [-1]), estimated as random effect. No Bonferroni correction was applied because there was only one dependent variable in this analysis. The model revealed that participants pressed the button more frequently on negative than on neutral trials, B = 0.69, 95% CI [0.21, 1.17], $p = .005$, $N = 58$. We conducted a similar model with reaction time (RT) in seconds as the outcome variable, which revealed no difference in speed with which people pressed the space bar on negative compared to neutral trials, B = -0.03, 95% CI [-0.33, 0.27], $p = .844$, $N = 46$. The valence contrast was estimated as random effect. Overall, the results of these analyses suggest that, in this particular experimental context, people used situation-targeted ER more frequently on negative relative to neutral trials; however, they were not faster to do so in high-arousal negative situations compared to low-arousal neutral situations.

Study 2 Preliminary Analyses

Did the picture stimuli have the intended emotional effect? To answer this question, we conducted two two-level models testing the effects of two valence contrasts on within-subjects (trial-by-trial) variation in emotional responding, as reflected in negative (NA) and positive affect (PA) rated at the end of each trial. Valence was represented by two different contrasts: positive versus negative (positive [1], neutral [0], negative [-1]) and emotional versus neutral (positive [1], neutral [-2], negative [1]). The valence contrasts were estimated as random effects that could, thus, vary between subjects. After applying the Bonferroni correction our critical p-value was .025.

The model using NA as the criterion variable revealed a significant effect of both valence contrasts. Participants reported greater NA in response to negative than positive pictures, B = -1.02, 95% CI [-1.11, -0.94], $p < .001$, and greater NA for emotional than neutral pictures, B = 0.36, 95% CI [0.33, 0.39], $p < .001$. To further understand this pattern, we applied model constraints to evaluate whether the differences between negative vs. neutral, and positive vs. neutral pictures were significant. Participants reported more NA for negative compared to neutral trials, B = 2.11, 95% CI [1.94, 2.27], $p < .001$, and no significant difference in NA for positive compared to neutral trials, B = 0.06, 95% CI [-0.00, 0.13], $p = .061$.

The model using PA as the criterion variable revealed that participants reported greater PA in response to positive than negative pictures, B = 0.83, 95% CI [0.75, 0.92], $p < .001$. There was no significant difference in PA for emotional compared to neutral pictures, B = 0.03, 95% CI [0, 0.06], $p = .094$. Additional model constraints as above for NA indicated that participants reported greater PA for positive compared to neutral pictures, B = 0.91, 95% CI [0.80, 1.02], $p < .001$. In addition, neutral pictures produced more PA compared to negative pictures, B = -0.76,

95% CI [-0.90, -0.61], $p < .001$. Overall, our analyses revealed that our stimuli elicited the desired emotional response.
